# Nutrition, Metabolites, and Human Health

**DOI:** 10.3390/nu15194286

**Published:** 2023-10-08

**Authors:** Christopher Papandreou

**Affiliations:** 1 Institute of Health Pere Virgili (IISPV), 43204 Reus, Spain; christoforos.papandreou@iispv.cat; 2 Nutrition and Metabolic Health Research Group, Department of Biochemistry and Biotechnology, Rovira i Virgili University (URV), 43201 Reus, Spain; 3 Center of Environmental, Food and Toxicological Technology—(TecnATox), Rovira i Virgili University, 43201 Reus, Spain; 4 Department of Nutrition and Dietetics Sciences, School of Health Sciences, Hellenic Mediterranean University (HMU), 72300 Siteia, Greece

The field of metabolomics and related “omics” techniques allows for the identification of a vast array of molecules within biospecimens. Their integration into nutritional studies may not only assist in the identification of potential nutritional biomarkers but also enhance our understanding of the biological mechanisms through which diet impacts health. This Special Issue of *Nutrients* entitled “Nutrition, Metabolites, and Human Health” adds to the current evidence from studies that have employed metabolomics and other omics techniques, either together with nutritional research or independently, to investigate their role in human health.

To explore the interplay between nutrition, metabolites and longevity, Li and colleagues examined the relationship between longevity-related metabolites and dietary nutrient intake profiles in Chinese Healthy centenarians and nonagenarians [1]. Untargeted metabolomics was used through ^1^H NMR spectroscopy in plasma and multivariate analysis revealed 13 metabolites associated with longevity, with enrichment in glycolysis/gluconeogenesis, aminoacyl-tRNA biosynthesis, and alanine, aspartate and glutamate metabolism pathways [1]. Further analysis indicated significant correlations between nutrient intake and plasma metabolites in long-lived people, offering insights into the interplay between nutrition, metabolism and longevity [1].

Cicalini and colleagues focused their study on the impact of maternal nutrition and lifestyle during pregnancy on newborns’ metabolic profiles [2]. Their findings showed correlations between physical activity, the use of iodized salt, supplement consumption, nervine drinks, smoking, and specific metabolites, expanding the scope of newborn screening to incorporate maternal lifestyle and dietary habits for enhanced understanding and diagnostic evaluation [2].

Considering the established link between postprandial metabolic dysregulations and disease risk, a comprehensive review paper in this issue sheds light on how metabolomics can improve our understanding of metabolic responses during nutritional challenges in humans [3]. The authors concluded that there are consistent findings among studies on the effects of Oral Glucose Tolerance Tests or mixed meals on key metabolic pathways governed by insulin action [3]. They also highlighted a decrease in phenotypic flexibility in individuals at increased cardiometabolic risk, which manifests as alterations in lipid and energy postprandial metabolism [3].

Utilizing data from the ZOE PREDICT 1 UK cohort, Bermingham and colleagues investigated inter-individual fasting and postprandial variabilities in metabolomic profiles assessed by NMR in healthy adults following sequential mixed meals [4]. Their findings indicated that postprandial responses for glycolysis, essential amino acids, ketone bodies and lipoprotein subclass particle plasma metabolites may offer more insight into metabolic responses and associated disease risk than fasting measures alone [4].

In the context of obesity, a study conducted in adults living with overweight and obesity examined associations between changes in circulating metabolites and changes in cardiometabolic risk factors during diet-induced weight loss (8 weeks) and weight loss maintenance (12 weeks) [5]. Decreases in circulating concentrations of phosphatidylcholines and sphingomyelins were associated with improvements in total cholesterol and low-density lipoprotein cholesterol levels during the low-calorie diet period. On the other hand, increases in these lipid species over the weight loss maintenance period were related to a deterioration in this lipid profile [5]. The authors concluded that changes in these lipid species in relation to changes in the lipid profile during weight loss and regain could help us to understand the potential role of metabolic responses in cardiovascular health [5].

This Special Issue also features three papers that focus on the relationship between gut-microbiota-derived or related metabolites and cardiometabolic health. In light of the concerning rise in childhood obesity, Śliżewska and colleagues demonstrated differences in the activity of fecal enzymes and profiles of fatty acids (short-chain fatty acids, branched-chain fatty acids) between obese children and those of normal weight. These results confirm the adverse metabolic effects associated with obesity, which can lead to the development of chronic diseases [6].

Galié and colleagues tried to unravel the network of microbial–host interactions and correlate it with cardiometabolic risk factors [7]. They identified a network of correlations between microbial genera and specific fecal and circulating metabolites that formed five distinct multi-omics clusters. These clusters were further associated with cardiometabolic risk factors, highlighting their importance in cardiometabolic health [7].

Additionally, in this Special Issue, in the area of microbial metabolites and cardiovascular health, a systematic review was conducted by Sanchez-Gimenez and colleagues on the role of gut-microbiota-derived metabolites in cardiovascular outcomes [8]. Their findings indicate inconsistent results for TMAO and bile acids but robust ones for the relationships between branched-chain amino acids and cardiovascular disease [8].

In this Special Issue, several studies using diverse “omics” approaches are discussed. Wang and colleagues explored the prospective associations of 25-hydroxyvitamin D [25(OH)D], parathyroid hormone (PTH), and β-C-terminal telopeptide of type 1 collagen (β-CTX) levels with all-cause mortality in centenarians [9]. Their findings revealed linear independent associations of 25(OH)D, while J-shaped associations were found between PTH and β-CTX levels and all-cause mortality [9]. When all these risk factors were combined, the effect size increased, highlighting the importance of PTH and β-CTX levels in the relationship between 25(OH)D insufficiency and mortality [9].

A protective role of long-term dietary docosahexaenoic acid (DHA) intake, which comes from fatty fish, against cognitive decline has been suggested by several epidemiological studies [10]. Sala-Vila and colleagues added to the growing body of evidence on the role of dietary factors in the risk for Alzheimer’s disease (AD) by examining associations between DHA measured in red blood cells (RBC) and AD and all-cause dementia in dementia-free participants aged ≥ 65 years old [11]. Individuals with the highest RBC DHA proportion had nearly half the risk of developing AD and all-cause dementia, and had an estimated 4.7 extra years of life free of AD compared to those with the lowest proportion. Interestingly, when analysis was stratified by APOE ε4 carrier status, a stronger association with dementia was observed in *ε*4 carriers [11].

In preterm neonates, necrotizing enterocolitis (NEC) is the most devastating gastrointestinal emergency, and research on early predictive biomarkers is fundamental. Moschino and colleagues performed a systematic review of studies applying untargeted metabolomics and gut microbiota analysis to evaluate the differences between neonates affected by NEC versus healthy controls [12]. Their research revealed differences in metabolomic profiles and microbiota diversity and composition between NEC-affected neonates and healthy controls, shedding light on potential biomarkers and the underlying pathophysiology of NEC [12].

Lately, the gut microbiota has attracted considerable attention due to their potential role in the treatment of obesity. Mo and colleagues conducted a randomized, double-blind, placebo-controlled study with 12-weeks probiotic supplementation in 72 individuals with overweight [13]. The anti-obesity effects of probiotics were observed by modulating the gut microbiota composition, which could lead to effective microbiota-targeted interventions.

On the other hand, the role of gut microbiota and metabolites in skin health is less clear. In a review article, Boyajian and colleagues summarized recent evidence on the relationship between the gut microbiome, cellular senescence and skin health [14].

Beyond human studies, in vivo and in vitro research was included in this Issue. Knutsdatter Østbye and collaborators explored the effects of camelina oil (CA), which is high in α-linolenic acid (ALA), and sandeel oil (SA), which is high in cetoleic acid, on the conversion of ALA to eicosapentaenoic acid (EPA) and DHA in obese rats [15]. The results suggest the active conversion of ALA to docosapentaenoic acid and DHA when a high amount of ALA was provided [15].

Lopez-Escalera and collaborators conducted an in vitro study to identify bacterial strains with potential prophylactic properties against non-alcoholic fatty liver disease [16]. They screened bacteria for their ability to strengthen the gut barrier, boost GLP-1 secretion, affect organoid transcriptomic profiles, and inhibit hepatic lipogenesis, with promising results for the B.lon_01 strain [16]. The results suggest that the B.lon_01 strain is a possible candidate, as it induced the highest increase in transepithelial electrical resistance and produced indole-3-lactic acid and indole-3-acetaldehyde, which are metabolites that may inhibit hepatic lipogenesis [16].

Another study by Chang et al. utilized a natural compound (β-lapachone) in combination with an aspartate aminotransferase inhibitor (aminooxyacetic acid) to assess their effects on metabolic perturbation in NQO1+ breast cancer breast cancer cells [17]. The authors suggested that this combinatorial treatment has synergistic effects on central metabolism, characterized by decreases in citrate, glutamate, and succinate enrichment, and could be a novel strategy to target breast cancer metabolic vulnerability [17].

In addition to the “omics” studies, this Special Issue features seven studies that explore various aspects of nutrition and health. Lin and collaborators examined the effects of individual-level and school-level factors on lipid profiles in adolescents from Taiwan [18]. An interplay between diet, physical activity, body mass index, health promotion programs, and food outlet density on lipid profiles was shown [18].

Beckmann and colleagues assessed the effects of a 12-week, school-based, multi-micronutrient supplementation (MMNS) and physical activity intervention in children from four South African primary schools, offering insights into the complexities of these interventions [19].

Wu and colleagues conducted a prospective study involving 1,516 adolescents, exploring longitudinal changes in the clustering of cardiometabolic parameters in adolescent metabolic syndrome (MetS) [20]. Their results shed light on the transformation of MetS status over time.

Agah and colleagues conducted a cross-sectional study involving Iranian adults to investigate the relationship between dietary macronutrients and dyspeptic symptoms [21]. They found that a higher fat or protein intake and lower carbohydrate intake were associated with a higher likelihood of experiencing uninvestigated dyspepsia [21].

In a retrospective study, Shi and collaborators examined clinical, biomedical, and pathological indicators able to distinguish risks between non-puerperal mastitis (NPM) and benign breast mass patients [22]. Their results suggested that dysregulations in lipid metabolism may play a pivotal role in the development of NPM [22].

Lee and collaborators investigated the effects of New Zealand spinach on andropause symptoms and lipid profiles in rats, revealing potential benefits [23].

Finally, Fu and colleagues explored the relationship between iodine status and mammary gland regulation in lactating female Wistar rats [24]. The study shed light on how the lactating mammary gland compensates for iodine deficiency and excess [24].

The Guest Editor of this Special Issue expresses his gratitude to all the contributing authors for their invaluable contributions. This Special Issue brings together studies ranging from cutting-edge ‘omics’ research to investigations into various aspects of health and nutrition. Hopefully, this research topic will not only advance our knowledge but also inspire further investigations.
